# Supplementary material for: Blood Transcript Biomarkers Selected by Machine Learning Algorithm Classify Neurodegenerative Diseases including Alzheimer’s Disease
Source: Biomolecules. 2022 Oct 29;12(11):1592. doi: 10.3390/biom12111592 (PMC9687215; doi:10.3390/biom12111592)
Supplement: Supplementary file 1 [file biomolecules-12-01592-s001.zip › biomolecules-1954691-supplementary.pdf]

Supplemental Materials

Blood transcript biomarkers selected by systematic machine learning algorithm classify neurodegenerative diseases including Alzheimer's disease.

Huseby, C.J., Delvaux, E., Brokaw, D., Coleman, P.D.

Keywords: linear discriminant analysis, multivariate analysis, machine learning, Random Forest classification, Alzheimer's Disease, Huntington's Disease, Parkinson's Disease, Amyotrophic lateral sclerosis or Lou Gehrig's disease, Frontotemporal dementia, Friedreich's ataxia, blood RNA biomarkers

	GEO dataset	description	Samples	Gender	platform	references
AD1	GSE63060<br>Nov 6, 2014<br>May 3, 2019	AD MCI HC<br>AddNeuroMed Cohort (batch 1)<br>Western European/Caucasian<br>Whole blood	329 samples:<br>104 HC<br>145 AD<br>80 MCI	 62F 42M<br>99F 46M<br>39F 41M	GPL6947 Illumina HumanHT-12 V3.0 expression beadchip	[1, 2]
AD2	GSE63061	AD MCI HC<br>AddNeuroMed Cohort (batch 2)<br>Western European/European mix<br>Whole blood	382 samples:<br>134 HC<br>139 AD<br>109 MCI	 81F 53M<br>85F 54M<br>65F 44M	GPL10558 Illumina HumanHT-12 V4.0 expression beadchip	[1]
PD1	GSE57475	PD HC<br>Blood a-synuclein, gene expression and smell testing as diagnostic and prognostic biomarkers in PD study from 22 US tertiary care centers.<br>Whole blood	142 samples<br>49 HC<br>93 PD – Dopamine transporter imaging confirmed.	 23F 26M<br>31F 62M	GPL6947 Illumina HumanHT-12 V3.0 expression beadchip	[3]
PD2<br><br>HD	GSE99039	PD HC HD other<br>GENEPARK consortium<br>Whole blood	558 samples:<br>**233 healthy control (HC)**<br>**205 idiopathic Parkinson's Disease (PD)**<br>**27 Huntington's Disease (HD)**<br>22 Genetic PD unaffected (GENUA)<br>41 Genetic PD affected (GPD)<br>30 MSA, PSP and other neurodegenerative disease	 142F 70M 21NA<br>90F 101M 14NA<br>11F 8M 8NA<br>8F 11M 3NA<br>19F 22M<br>11F 12M 7NA	GPL570 (HG-U133_Plus_2) Affymetrix Human Genome U133 Plus 2.0 Array	[4]
ALS1	GSE112676	ALS HC<br>Tertiary referral center for motor neuron diseases University Medical Center Utrecht, The Netherlands.<br>Whole blood at diagnosis	741 samples:<br>508 HC<br>233 ALS<br>143 spinal<br>90 bulbar	 230F 278M<br>90F 143M<br>48F 95M<br>42F 48M	GPL6947 Illumina HumanHT-12 V3.0 expression beadchip	[5, 6]
ALS2	GSE112680	ALS HC MIMICS<br>Whole blood	376 samples:<br>137 HC<br>164 ALS<br>108 spinal<br>56 bulbar<br>75 MIMICS	 58F 79M<br>68F 96M<br>31F 77M<br>37F 19M<br>17F 58M	Illumina GPL10558 HumanHT-12 V4.0 expression beadchip	[5, 6]
FRDA	GSE102008	FRDA HC CARRIERS<br>UCLA and Children's Hospital of Philadelphia<br>Whole blood	733 samples:<br>94 HC<br>411 FRDA<br>228 CARRIERS	 40F 54M<br>192F 219M<br>141F 87M	Illumina GPL10558 HumanHT-12 V4.0 expression beadchip	[7, 8]
FTA	GSE140830	bvFTD HC other dementia<br>UCLA, UCSF<br>Whole blood	542 samples<br>281 HC<br>80 bvFTD<br>47 nfvPPA<br>54 PSP<br>44 svPPA<br>36 CBS	 156F 125 M<br>35F 45M<br>28F 19M<br>29F 25M<br>21F 23M<br>20F 16M	GPL15988 Illumina HumanHT-12 V4.0 expression beadchip<br>nuID	[9]; Nachun, D. etal., 2019
AD3	GSE140829	AD MCI HC<br>UCLA, UCSF<br>Whole blood	587 samples<br>249 HC<br>204 AD<br>134 MCI	 104F 100M<br>139F 110 M<br>62F 72M	GPL15988 Illumina HumanHT-12 V4.0 expression beadchip<br>nuID	[9]; Nachun, D. etal., 2019

Table S1. Characteristics of RNA expression data sets used in this study. HC-health[3, 9]y control, AD-Alzheimer's disease, MCI-mild cognitive impairment, PD-Parkinsons's disease, ALS- amyotrophic lateral sclerosis, MIMIC-diseases mimicking ALS, FRDA-Friedreich's ataxia, CARRIERS-heterozygous unaffected carriers of FRDA, bvFTD-behavioral variant frontotemporal dementia, nfvPPA- _____________, PSP-_______________, svPPA-____________, CBS-______________. Dates reflect data submission to GEO and last update since November 24, 2020.

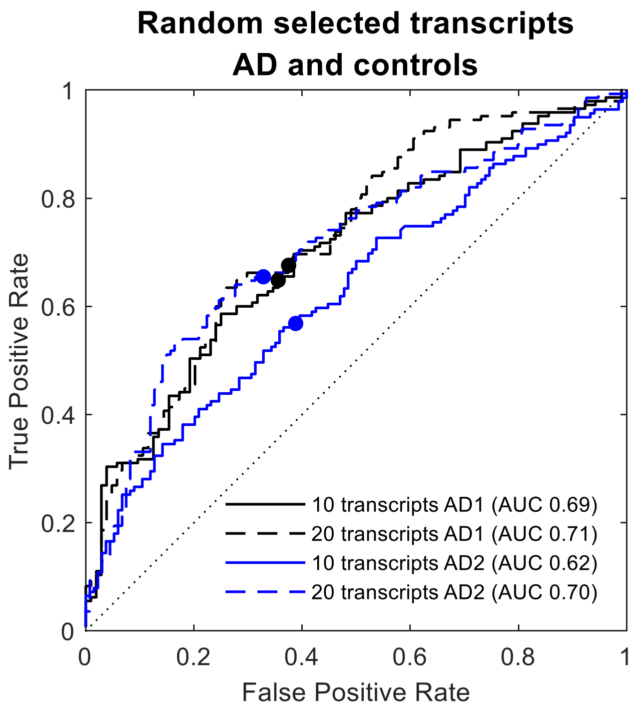

Figure S1 Randomly selected transcripts performance in data. ROC for randomly generated set of 20 transcripts and a subset of 10 of those transcripts were tested on GSE63060 AD1 (black) and GSE63061 AD2 (blue). AUCs, calculated using discriminant scores for LDA. Dot marks LDA selected threshold for classifiction of disease from cotnrols.

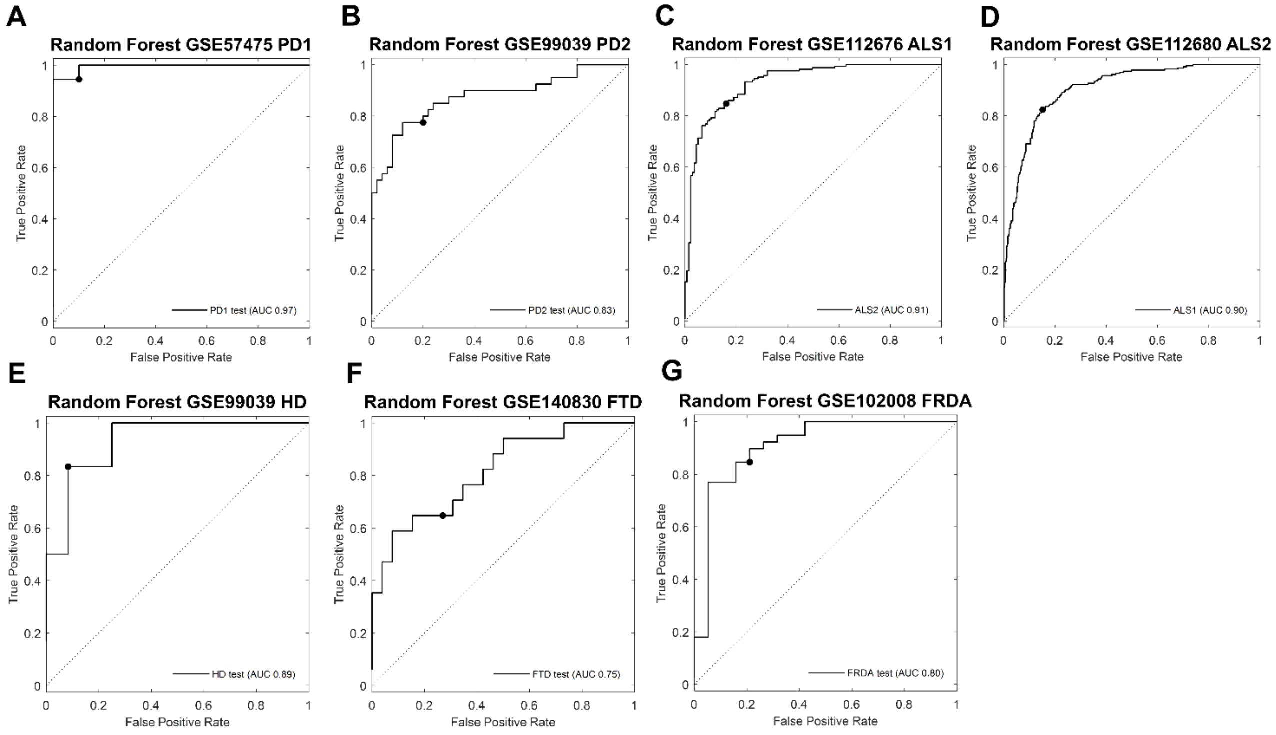

Figure S2 Random forest other neurodegenerative diseases.

A) Top 20 Random forest transcript picks to select PD from controls. Trained and tested within GSE57475. Model# 71 was selected with sensitivity 94%. Receiver operator curves of discriminant scores generated from Linear discriminant analysis. LDA midpoint cutoff indicated on curves by solid dot. **B)** Top 20 Random forest transcript picks to select PD from controls. Trained and tested within GSE99039. Model #74 was selected with sensitivity 60%. Receiver operator curves of discriminant scores generated from Linear discriminant analysis. LDA midpoint cutoff indicated on curves by solid dot. **C)** Top 20 Random forest transcript picks to select other neurodegenerative diseases from controls. Trained on GSE112676 and tested on GSE112680. Model #33 was selected with sensitivity 74%. Receiver operator curves of discriminant scores generated from Linear discriminant analysis. LDA midpoint cutoff indicated on curves by solid dot. **D)** Top 20 Random forest transcript picks to select ALS from controls. Trained on GSE112680 and tested on GSE112676. Model #98 was selected with sensitivity 91%. Receiver operator curves of discriminant scores generated from Linear discriminant analysis. LDA midpoint cutoff indicated on curves by solid dot. **E)** Top 20 Random forest transcript picks to select HD from controls. Trained and tested within GSE99039. Model #48 was selected with sensitivity 83%. Because the number of transcripts cannot be larger than the number of samples in the test set (18 test samples), receiver operator curves of discriminant scores generated from Linear discriminant analysis performed on the top 10 transcripts only. LDA midpoint cutoff indicated on curves by solid dot. **F)** Top 20 Random forest transcript picks to select FTD from controls. Trained and tested within GSE140830. Model #25 was selected with sensitivity 71%. Receiver operator curves of discriminant scores generated from Linear discriminant analysis. LDA midpoint cutoff indicated on curves by solid dot. **G)** Top 20 Random forest transcript picks to select FRDA from controls. Trained and tested within GSE102008. Model #79 with sensitivity 79%. Receiver operator curves of discriminant scores generated from Linear discriminant analysis. LDA midpoint cutoff indicated on curves by solid dot.

Table S2 LDA statistics for empirically selected transcripts

2017	Inflammation	Epigenetics	Stress	All	Inflammation	Epigenetics	Stress	All
	GSE63060 AD				GSE63061 AD			
Wilks' Λ	0.8425	0.9069	0.8976	0.6957	0.9550	0.9575	0.9433	0.8552
DoF	7/241	6/242	9/239	22/226	7/265	6/266	9/263	22/250
p-value	1.0E-04	5.6E-04	1.9E-03	1.0E-04	9.1E-02	7.1E-02	7.7E-02	9.1E-03
Correct	66.67%	62.25%	64.26%	74.70%	57.88%	58.97%	60.81%	68.13%
sensitivity	64.83%	64.83%	64.42%	76.55%	57.55%	61.87%	59.71%	68.35%
specificity	69.23%	58.65%	64.14%	72.12%	58.21%	55.97%	61.94%	67.91%
AUC	0.72	0.65	0.66	0.80	0.61	0.60	0.62	0.72
	GSE112676 ALS				GSE112680 ALS			
Wilks' Λ	0.8702	0.8547	0.7740	0.7443	0.9259	0.8333	0.8151	0.6736
DoF	7/733	5/735	6/734	18/722	7/293	5/295	6/294	18/282
p-value	1.0E-04	1.0E-04	1.0E-04	1.0E-04	1.9E-03	1.0E-04	1.0E-04	1.0E-04
Correct	67.61%	69.23%	72.87%	73.95%	60.8%	66.78%	68.44%	76.74%
sensitivity	55.36%	57.94%	60.94%	63.52%	58.54%	65.85%	66.46%	73.17%
specificity	73.23%	74.41%	78.35%	78.74%	63.5%	67.88%	70.8%	81.02%
AUC	0.71	0.72	0.77	0.79	0.65	0.72	0.75	0.80
	GSE57475 PD				GSE99039 PD			
Wilks' Λ	0.9396	0.9807	0.9269	0.8635	0.9554	0.9794	0.9347	0.8884
DoF	7/134	6/135	9/132	22/119	7/429	6/430	9/427	22/414
p-value	2.9E-01	8.5E-01	3.3E-01	6.5E-01	6.3E-03	1.7E-01	6.4E-04	5.6E-04
Correct	59.86%	59.86%	59.86%	68.31%	60.18%	55.84%	60.64%	62.7%
sensitivity	62.37%	63.44%	59.14%	72.04%	61.46%	53.17%	58.05%	60.49%
specificity	55.1%	53.06%	61.22%	61.22%	59.05%	58.19%	62.93%	64.66%
AUC	0.60	0.55	0.61	0.67	0.61	0.58	0.64	0.67
	GSE140830 bvFTD				GSE102008 FRDA			
Wilks' Λ	0.9554	0.9716	0.9683	0.9095	0.9772	0.9766	0.9750	0.9284
DoF	7/353	6/354	9/351	22/338	7/497	6/498	9/495	22/482
p-value	2.3E-02	1.1E-01	2.5E-01	6.2E-02	1.2E-01	6.5E-02	1.8E-01	2.6E-02
Correct	57.62%	56.51%	59.28%	63.16%	59.21%	59.21%	58.42%	64.95%
sensitivity	63.75%	60.0%	57.5%	60.0%	60.34%	59.12%	57.91%	63.26%
specificity	55.87%	55.52%	59.79%	64.06%	54.26%	59.57%	60.64%	72.34%
AUC	0.63	0.61	0.62	0.70	0.58	0.60	0.60	0.68
	GSE99039 HD				GSE140829 AD			
Wilks' Λ	0.9359	0.9559	0.8791	0.8621	0.9574	0.9576	0.9352	0.8803
DoF	7/251	6/252	9/249	22/236	7/445	6/446	9/443	22/430
p-value	1.9E-021	7.5E-02	1.6E-04	2.7E-02	6.8E-03	3.5E-03	4.5E-04	1.0E-04
Correct	69.5%	62.93%	76.83%	74.52%	57.17%	56.29%	62.03%	64.46%
sensitivity	59.26%	66.67%	70.37%	66.67%	56.86%	55.88%	65.69%	63.73%
specificity	70.69%	62.5%	77.59%	75.43%	57.43%	56.63%	59.04%	65.06%
AUC	0.68	0.67	0.75	0.75	--	--	--	--

DoF - degrees of freedom; AUC - area under curve; Correct - percent samples correctly classified; AD – Alzheimer's disease; bvFTD – behavioral variant frontotemporal dementia; ALS – Amyotrophic lateral sclerosis; FRDA – Friedreich's ataxia; PD – Parkinson's disease; HD – Huntington's disease

Table S3 LDA statistics Random Forest AD GSE63060 and GSE63061

Random Forest	Training on GSE63060	Training on GSE63061	Training on GSE63060	Training on GSE63061
	GSE63060 AD		GSE63061 AD	
Wilks' Λ		0.5509	0.6732	
DoF		20/228	20/252	
p-value		1.0E-04	1.0E-04	
Correct		83.13%	75.09%	
sensitivity		84.83%	71.22%	
specificity		80.77%	79.1%	
AUC		0.87	0.82	
	GSE112676 ALS		GSE112680 ALS	
Wilks' Λ	0.7218	0.8680	0.5715	0.8667
DoF	16/724	9/734	16/284	9/294
p-value	1.0E-04	1.0E-04	1.0E-04	1.0E-04
Correct	75.44%	66.4%	81.4%	68.11%
sensitivity	76.82%	63.09%	84.76%	67.07%
specificity	74.8%	67.91%	77.37%	69.34
AUC	0.83	0.71	0.87	0.70
	GSE57475 PD		GSE99039 PD	
Wilks' Λ	0.8751	0.9111	0.9140	0.9070
DoF	20/121	15/126	18/418	12/424
p-value	6.3E-01	6.5E-01	3.6E-03	1.0E-04
Correct	69.01%	61.97%	63.84%	64.53%
sensitivity	74.19%	63.44%	61.46%	61.95%
specificity	59.18%	59.18%	65.95%	66.81%
AUC	0.67	0.62	0.67	0.67
	GSE140830 bvFTD		GSE102008 FRDA	
Wilks' Λ	0.9041	0.8836	0.9518	0.9465
DoF	20/340	20/340	19/485	17/487
p-value	1.9E-02	1.9E-03	1.8E-01	5.5E-02
Correct	64.27%	64.54%	61.58%	60.4%
sensitivity	65.0%	71.25%	62.04%	61.56%
specificity	64.04%	62.63%	59.57%	55.32%
AUC	0.70	0.74	0.63	0.63
	GSE99039 HD		GSE140829 AD	
Wilks' Λ	0.806	0.9072	0.8522	0.8484
DoF	18/240	11/247	20/432	20/432
p-value	1.0E-04	1.1E-02	1.0E-04	1.0E-04
Correct	79.15%	76.06%	66.45%	68.6%5
sensitivity	62.96%	74.07%	69.61%	71.08%
specificity	81.03%	76.29%	63.86%	66.67%
AUC	0.83	0.80	71%	72%

DoF - degrees of freedom; AUC - area under curve; Correct - percent samples correctly classified; AD – Alzheimer's disease; bvFTD – behavioral variant frontotemporal dementia; ALS – Amyotrophic lateral sclerosis; FRDA – Friedreich's ataxia; PD – Parkinson's disease; HD – Huntington's disease

Table S4 LDA statistics Random forest transcripts other neurodegenerative diseases.

	Train on	Train on	Training set 80% and Test set 20%				
**Random forest**	**GSE112676 ALS**	**GSE112680 ALS**	**GSE57475 PD**	**GSE99039 PD**	**GSE99039 HD**	**GSE140830 bvFTD**	**GSE102008 FRDA**
**Wilks' Λ**	0.5011	0.5757	0.1297	0.5983	0.4884	0.6958	0.5483
**DoF**	20/280	20/720	20/7	20/69	10/7	20/22	20/37
**p-value**	1.0E-04	1.0E-04	1.3E-01	5.4E-03	6.8E-01	9.5E-01	1.3E-01
**Correct**	84.39%	84.08%	100%	78.89%	88.89%	69.77%	82.76%
**sensitivity**	84.76%	82.4%	100%	77.5%	83.33%	64.71%	84.62%
**specificity**	83.94%	84.84%	100%	80.0%	91.67%	73.08%	78.95%
**AUC**	0.91	0.90	0.97	0.83	0.89	0.75	0.80

DoF - degrees of freedom; AUC - area under curve; Correct - percent samples correctly classified; bvFTD – behavioral variant frontotemporal dementia; ALS – Amyotrophic lateral sclerosis; FRDA – Friedreich's ataxia; PD – Parkinson's disease; HD – Huntington's disease

References

1. Sood, S., et al., *A novel multi-tissue RNA diagnostic of healthy ageing relates to cognitive health status.* Genome Biol, 2015. **16**: p. 185.
2. Lunnon, K., et al., *Mitochondrial dysfunction and immune activation are detectable in early Alzheimer's disease blood.* J Alzheimers Dis, 2012. **30**(3): p. 685-710.
3. Locascio, J.J., et al., *Association between alpha-synuclein blood transcripts and early, neuroimaging-supported Parkinson's disease.* Brain, 2015. **138**(Pt 9): p. 2659-71.
4. Shamir, R., et al., *Analysis of blood-based gene expression in idiopathic Parkinson disease.* Neurology, 2017. **89**(16): p. 1676-1683.
5. van Rheenen, W., et al., *Whole blood transcriptome analysis in amyotrophic lateral sclerosis: A biomarker study.* PLoS One, 2018. **13**(6): p. e0198874.
6. Swindell, W.R., et al., *ALS blood expression profiling identifies new biomarkers, patient subgroups, and evidence for neutrophilia and hypoxia.* J Transl Med, 2019. **17**(1): p. 170.
7. Nachun, D., et al., *Peripheral blood gene expression reveals an inflammatory transcriptomic signature in Friedreich's ataxia patients.* Hum Mol Genet, 2018. **27**(17): p. 2965-2977.
8. Lai, J.I., et al., *Transcriptional profiling of isogenic Friedreich ataxia neurons and effect of an HDAC inhibitor on disease signatures.* J Biol Chem, 2019. **294**(6): p. 1846-1859.
9. Du, P., W.A. Kibbe, and S.M. Lin, *nuID: a universal naming scheme of oligonucleotides for illumina, affymetrix, and other microarrays.* Biol Direct, 2007. **2**: p. 16.
